# Dissecting molecular mechanisms underlying H_2_O_2_-induced apoptosis of mouse bone marrow mesenchymal stem cell: role of Mst1 inhibition

**DOI:** 10.1186/s13287-020-02041-7

**Published:** 2020-12-09

**Authors:** Qian Zhang, Xianfeng Cheng, Haizhou Zhang, Tao Zhang, Zhengjun Wang, Wenlong Zhang, Wancheng Yu

**Affiliations:** 1 Department of Cardiovascular Surgery, Shandong Provincial Hospital Affiliated to Shandong First Medical University, Jinan, 250062 Shandong China; 2 Department of Cardiovascular Surgery, Weifang People’s Hospital, Weifang, 261000 Shandong China

**Keywords:** Mst1, Bone marrow mesenchymal stem cell, Reactive oxygen species, Cell apoptosis, Autophagy

## Abstract

**Background:**

Bone marrow mesenchymal stem cell (BM-MSC) has been shown to treat pulmonary arterial hypertension (PAH). However, excessive reactive oxygen species (ROS) increases the apoptosis of BM-MSCs, leading to poor survival and engraft efficiency. Thus, improving the ability of BM-MSCs to scavenge ROS may considerably enhance the effectiveness of transplantation therapy. Mammalian Ste20-like kinase 1 (Mst1) is a pro-apoptotic molecule which increases ROS production. The aim of this study is to uncover the underlying mechanisms the effect of Mst1 inhibition on the tolerance of BM-MSCs under H_2_O_2_ condition.

**Methods:**

Mst1 expression in BM-MSCs was inhibited via transfection with adenoviruses expressing a short hairpin (sh) RNA directed against Mst1 (Ad-sh-Mst1) and exposure to H_2_O_2_. Cell viability was detected by Cell Counting Kit 8 (CCK-8) assay, and cell apoptosis was analyzed by Annexin V-FITC/PI, Caspase 3 Activity Assay kits, and pro caspase 3 expression. ROS level was evaluated by the ROS probe DCFH-DA, mitochondrial membrane potential (ΔΨm) assay, SOD1/2, CAT, and GPx expression. Autophagy was assessed using transmission electron microscopy, stubRFP-sensGFP-LC3 lentivirus, and autophagy-related protein expression. The autophagy/Keap1/Nrf2 signal in H_2_O_2_-treated BM-MSC/sh-Mst1 was also measured.

**Results:**

Mst1 inhibition reduced ROS production; increased antioxidant enzyme SOD1/2, CAT, and GPx expression; maintained ΔΨm; and alleviated cell apoptosis in H_2_O_2_-treated BM-MSCs. In addition, this phenomenon was closely correlated with the autophagy/Keap1/Nrf2 signal pathway. Moreover, the antioxidant pathway Keap1/Nrf2 was also blocked when autophagy was inhibited by the autophagy inhibitor 3-MA. However, Keap1 or Nrf2 knockout via siRNA had no effect on autophagy activation or suppression.

**Conclusion:**

Mst1 inhibition mediated the cytoprotective action of mBM-MSCs against H_2_O_2_-induced oxidative stress injury. The underlying mechanisms involve autophagy activation and the Keap1/Nrf2 signal pathway.

**Graphical abstract:**

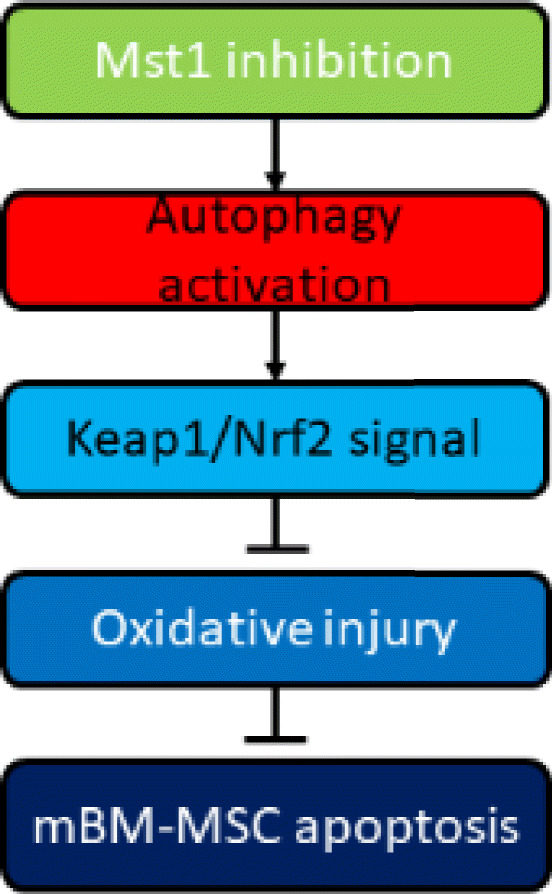

**Supplementary information:**

**Supplementary information** accompanies this paper at 10.1186/s13287-020-02041-7.

## Significance statement

In the current study, the authors validate for the first time that Mst1 inhibition reduced ROS production; increased antioxidant enzyme SOD1/2, CAT, and GPx expression; maintained ΔΨm; and alleviated cell apoptosis in H_2_O_2_-treated BM-MSCs. In addition, this phenomenon was closely correlated with the autophagy/Keap1/Nrf2 signal pathway. The autophagy inhibitor, the antioxidant pathway Keap1/Nrf2, was also blocked when autophagy was inhibited by 3-MA. However, Keap1 or Nrf2 knockout via siRNA had no effect on autophagy activation or suppression. These findings present the efficient protective capacity of transplanted MSCs in PAH.

## Introduction

Mesenchymal stem cell (MSC)-based therapies have been investigated for pulmonary arterial hypertension (PAH) treatment due to the “homing” ability [1, 2]. Increasing evidence supports the enhanced generation of pathological levels of reactive oxygen species (ROS) at the injured pulmonary vasculature in PAH [3]. MSCs can adjust with oxidative stress, and low level ROS regulates the self-renewal of stem cells [4]. However, MSC apoptosis increases dramatically when the ROS level exceeds the basal level [5]. The excessive ROS at the injury sites and the loss of transplanted MSCs from these sites are correlated [6]. Excessive ROS induces MSC apoptosis, leading to poor survival and engraft efficiency [7, 8]. Thus, improving the ROS scavenging ability of MSCs is essential to promote MSC engraftment and enhance tissue repair.

Mammalian Ste20-like kinase 1 (Mst1) is an ubiquitously expressed serine/threonine kinase and a component of the Hippo signaling pathway, which regulates cell apoptosis, proliferation, and organ size [9]. Mst1 is well known as a pro-apoptotic molecule, and its suppression alleviates cell apoptosis by decreasing ROS production [10]. Mst1 was recently found to act as a switch that simultaneously regulates apoptosis and autophagy in cardiomyocytes [11, 12]. Autophagy is an evolutionary process that recycles cellular components and damaged organelles in response to oxidative stress. In this process, autophagy, in turn, may contribute to reduce oxidative damages by removing ROS byproduct [13] and has a prosurvival role in stem cells, the downregulation of which results in rapid cell death [14]. To date, no study has identified the role of Mst1 in oxidative stress-induced cell apoptosis in MSCs.

Evidence has showed that Mst1 inhibition maintains cellular redox balance by modulating Nrf2 with the help of Keap1 [15]. The Keap1/Nrf2 signal pathway was demonstrated as the major node of cellular defense against oxidative stress. Keap1 acts as a sensor of redox insults, and under quiescent conditions, the cytoplasmic protein Keap1 usually binds Nrf2 and prevents its translocation to the nucleus. During oxidative stress, Nrf2 is de-repressed and activates a battery of cytoprotective genes, such as glutathione peroxidase (GPx), superoxide dismutase (SOD), and Catalase (CAT) [16, 17]. Besides, among all MSC types, bone marrow mesenchymal stem cells (BM-MSCs) are the adult stem cells that show potential for tissue regeneration through its self-renewal ability [18]. In previous study, thus, our hypothesis was that Mst1 inhibition enhanced the tolerance of BM-MSCs under H_2_O_2_ condition. To prove our hypothesis, we investigate the modulation of mouse BM-MSCs (mBM-MSCs) via Mst1 expression downregulation under H_2_O_2_ conditions and determine its underlying mechanisms. This work focused on mediating autophagy and Keap1/Nrf2 signaling pathway.

## Methods

### mBM-MSC culture

In this research, all animal experimental protocols were approved by the Animal Care Committee of School of Medicine, Shandong First Medical University. mBM-MSCs were isolated from 8-week-old C57BL/6 mice as described previously (see Supporting Information Methods) [19]. After 48 h, the cultures were washed with PBS to remove the non-adherent cells, harvested, and expanded in 175 cm^2^ flasks until > 80% confluence. This batch was designated as passage 1. Cultured mBM-MSCs between passages 3 and 5 were used for the following experiments.

### Identification of mBM-MSCs

The identification and characterization method of isolated mBM-MSCs were presented as previously described [20]. The cultured mBM-MSCs (1 × 10^6^/mL) were stained with the following antibodies (Supporting Information Table S1) and analyzed by flow cytometry (Guava easyCyte 6 HT, Merk Millipore, USA). The results were analyzed by GuavaSoft 3.1.1.

Phenotypic characterization and differentiation into osteoblasts, adipocytes, and chondrocytes were tested using Mouse Mesenchymal Stem Cell (mMSC) Osteogenic Differentiation Complete medium, mMSC adipocyte Induction And Differentiation Complete medium, and mMSC Chondrocyte Differentiation Complete medium (Cyagen Biosciences Inc., China), respectively, as the manufacturer’s instructions described. Staining was visualized by light microscopy.

### Adenovirus and siRNA constructs

Adenoviruses expressing a short hairpin (sh) RNA directed against Mst1 (Ad-sh-Mst1) and harboring control vectors for Ad-sh-Mst1 (Ad-LacZ) were constructed by Genechem Co., Ltd. (China) and transduced to BM-MSCs 24 h after transduction of stubRFP-sensGFP-LC3 lentivirus as the instruction described. The titers of adenoviruses were 1 × 10^10^ PFU/ml. The multiplicity of infection used was 100:1. The shRNA sequence targeting mouse Mst1 is shown in Supporting Information Table S2.

Nrf2 and Keap1 siRNAs were obtained from GenePharma Co., Ltd. (China). Nrf2-siRNA, Keap1-siRNA, and scrambled siRNA (siCTL) are shown in Supporting Information Table S2. siCTL were used as the control. siRNAs (50 nM) were transfected into cells by using the Lipofectamine RNAi MAX (Invitrogen). The transfected cells were used for subsequent experiments after incubation for 12 h at 37 °C.

### Cell treatment

For mBM-MSC transduction, cells growing at an exponential phase were randomly divided into following groups: control group (only mBM-MSCs), model groups (mBM-MSCs treated with 200 μM of H_2_O_2_ for 12 h), negative group (mBM-MSCs transduced with Ad-LacZ and treated with 200 μM of H_2_O_2_ for 12 h), sh-Mst1 group (mBM-MSCs transduced with Ad-sh-Mst1 and then treated with 200 μM of H_2_O_2_ for 12 h), and sh-Mst1+3-MA group (mBM-MSCs were transduced with Ad-sh-Mst1, pretreated with 5 mM 3-methyladenine or 3-MA from Selleck, USA, for 45 min, and treated with 200 μM of H_2_O_2_ for 12 h), sh-Mst1+ si-ctrl group (mBM-MSCs transduced with Ad-sh-Mst1, transfected with control-siRNA, followed by treatment with 200 μM H_2_O_2_ for 12 h), and sh-Mst1+si-Nrf2 group (mBM-MSCs transduced with Ad-sh-Mst1, transfected with Nrf2-siRNA, followed by treatment with 200 μM H_2_O_2_ for 12 h).

### Cell adhesion

Cell adhesion was measured using Matrigel (BD) as performed previously (see Supporting Information Methods) [21]. Optical density (OD) was measured at 590 nm using Multiskan MK3 microplate reader. The experiment was repeated three times. Cell adhesion was calculated as ratio of control.

### Cell Counting Kit 8 (CCK-8) assay

The cells (3 × 10^3^) were seeded in 96-well plates. Following experimental treatment, 10 μl α-MEM with CCK-8 (Beyotime Institute of Biotechnology, China) was added in accordance with the manufacturer’s protocol. OD of the well was measured at 450 nm by using a Multiskan MK3 microplate reader and experiments were repeated three times. Cell viability was calculated as ratio of control.

### Transferase-mediated dUTP nick-end labeling (TUNEL) method

Cell apoptosis was analyzed with a one-step TUNEL kit in accordance with the manufacturer’s instructions (Beyotime). In brief, cells were permeabilized with 0.1% Triton X-100 for 2 min on ice followed by TUNEL for 1 h at room temperature. TUNEL-positive cells were measured at 550-nm excitation and 570-nm emission by fluorescence microscopy (CKX71, Olympus). The cells with red fluorescence were identified as apoptotic cells.

### Annexin V-fluorescein isothiocyanate (FITC)/propidium iodide (PI) assay

The apoptotic level was analyzed using an Annexin V-FITC Apoptosis Detection kit (Beyotime) as described by Ezzatollah Fathi [22]. In brief, mBM-MSCs (5 × 10^5^ cells) were harvested after the appropriate treatments and washed with the complete medium. Then, cells were suspended in 195 μL of binding buffer and incubated with 5 μL of Annexin V-FITC and 5 μL of PI at 25 °C for 15 min in the dark. The samples were studied by flow cytometry (Becton, Dickinson) and data were analyzed using the BD FACSDiva software.

### Caspase 3 activity assay

Caspase 3 activity was measured using the Caspase 3 Activity Assay kit (Beyotime) according to the manufacturer’s protocol. Briefly, cells (5 × 10^4^ cells) and culture medium were collected and centrifuged at 4 °C, 600*g* for 5 min, then resuspended with 50 μl cell lysis buffer, and incubated on ice for 15 min before being centrifuged at 4 °C, 18,000*g* for 10 min. Cell lysate supernatant was collected. Forty-microliter assay buffer (supplied with the kit), 50 μl cell lysate supernatant, and the 10 μl Caspase 3 substrate Ac-DEVD-pNA (2 mM) were combined and incubated at 37 °C for 120 min, and then, Caspase 3 activity was measured at 405 nm using Multiskan MK3 microplate reader. The relative caspase3 activity was calculated as the ratio of control group. The assay was repeated 3 times.

### Measurement of reactive oxidative species (ROS)

Cellular ROS was assessed via the ROS probe DCFH-DA (Beyotime Biotechnology, China). After treatment, cells were washed with PBS and incubated with a DCFH-DA probe (10 μM) at 37 °C for 10 min. Then, PBS was used to remove the free ROS probe. The mean fluorescence intensity was detected via flow cytometry.

### Measurement of the mitochondrial membrane potential (Δψm)

Δψm was evaluated via JC-1 staining (Beyotime). In short, cells were plated (5 × 10^4^ cells/mL) in six wells and after appropriate treatments, treated with 5 mg/mL of JC-1 probe at 37 °C for 15 min, and washed with PBS to remove the free JC-1 probe. Images were taken by using fluorescent microscopy. The ratio (%) of fluorescence red/green fluorescence intensity was calculated by Image J software, and the value was calculated relative to that of the control group.

### Transmission electron microscopy (TEM)

The cells were fixed in 3% glutaraldehyde at 4 °C for 2 h, post-fixed, dehydrated, cut, and stained. Finally, the images were captured using a JEM-1200EX transmission electron microscope (Japan Electronics and Optics Laboratory, Tokyo, Japan). At least three randomly selected areas were obtained, and the EM assay was repeated three times.

### Autophagy detection

The autophagy indicator, stubRFP-sensGFP-LC3 lentivirus, was purchased from Hanbio Biotechnology Co., Ltd. (Shanghai, China). In brief, cells were infected with the autophagy indicator for 24 h. The medium was replaced completely with a new one. After 5 days of transfection, cells were transduced with adenoviruses expressing vectors and treated with H_2_O_2_. Finally, autophagosomes (yellow dots) and autolysosome (red dots) were detected under a confocal microscope to detect the autophagy flux.

### Real-time PCR

Total RNA was extracted from BM-MSCs using a Trizol reagent (Tiangen Biotech Co., Ltd., China). Then, 1 μg of RNA was converted to cDNA and amplified the aimed gene fragment detected with SYBR Green qPCR kit (TOYOBO, Japan). qPCR was performed for 40 cycles. The relative gene expression was normalized to the expression of GAPDH, a housekeeping gene, through the 2^−△△CT^ method. Three independent replicates were performed to verify the reproducibility of data. Primers were purchased from Sangon Biological Engineering Technology & Services (Shanghai, China). The sequences for Mst1 are shown in Supporting Information Table S3.

### Western blot analysis

Proteins were extracted from the treated cells by using ice cold lysis buffer containing phenylmethylsulfonyl fluoride. Protein content was determined with a bicinchoninic acid protein assay kit (Beyotime) using bovine serum albumin as the standard. Equal amounts of protein (15 μg) were loaded onto 10% SDS-PAGE and transferred to a PVDF membrane (Millipore Corp, Billerica, MA, USA) through the wet transfer method. The membranes were then blocked for 1 h with 5% skimmed milk or BSA in TBST and incubated overnight at 4 °C with the following primary antibodies (diluted by Western Primary Antibody Buffer, Beyotime) (see Supporting Information Table S1). β-actin served as the loading control. The PVDF membranes were incubated for 1 h with secondary goat anti-rabbit IgG-conjugated HRP (1:1000, Beyotime). Protein bands were visualized using Millipore Immobion™ Western Chemiluminescent HRP Substrate (Millipore). The resulting band intensity was quantified with software ImageJ.

### Statistical analysis

SPSS 16.0 statistical package (Chicago, IL, USA) was used for data analysis. The qualitative data were compared with Fisher’s exact test. The values were expressed as means ± standard deviation (SD). The statistical significance of difference was calculated by one-way ANOVA followed by the post hoc tests of Tukey. *p* < 0.05 was considered statistically significant.

## Results

### mBM-MSC characterization

**Fig. 1:**
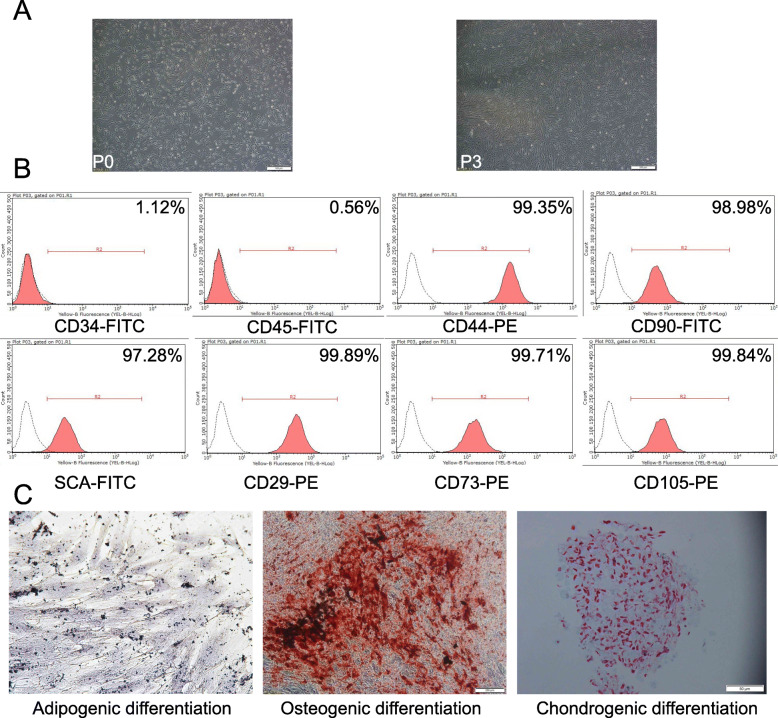
Identity of mBM-MSCs. **a** mBM-MSC culture morphology. **b** Representative flow cytometry analysis of mBM-MSCs. Positive expression of MSC markers (CD44, CD90, Sca-1, CD29, CD73, and CD105) and absence of hematopoietic markers (CD45 and CD34). **c** Differentiation potential of mBM-MSCs. mBM-MSCs were tested for their ability to differentiate into adipocytes (stained with Oil red O), osteoblasts (stained with alkaline phosphatase), and chondrocytes (stained with Safranin O-Fast Green). The representative images are presented here

After 7–13 days of culture, the isolated mBM-MSCs reached 80% confluence and were passaged. Cell morphology was examined under an inverted microscope (Fig. 1a). Passage 0 (P0) displayed various shapes: protrusions from the edges, polygonal, long fusiform, and irregular shapes. P3 cells were formed with cells displaying a spindle shape and arranged in radial concentric circles or with broom-like growth. The uniformity of the passaged cells was enhanced, resulting in their long-spindle shape. Cells at passage 3 were examined by flow cytometry using surface markers (Fig. 1b). The isolated mBM-MSCs were negative for CD34 (1.12%) and CD45 (0.56%) but positive for CD44 (99.35%), CD90 (98.98%), SCA-1 (97.28%), CD29 (99.89%), CD73 (99.71%), and CD105 (99.84%). Moreover, the differentiation potential of mBM-MSCs was identified (Fig. 1c). After culturing with adipogenic induction and differentiation medium for 21 days, osteogenic differentiation medium for 28 days, or chondrogenic induction medium for 28 days, the cells were positively stained by Oil red O, alkaline phosphatase, or Safranin O-Fast Green, respectively. The results indicated that the isolated mBM-MSCs can be used for the subsequent experiments.

### Mst1 inhibition changed the biological properties of H_2_O_2_-treated mBM-MSCs

**Fig. 2:**
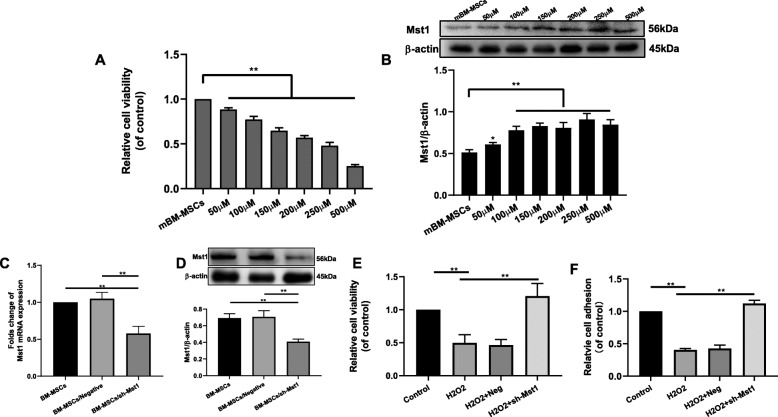
Expression of Mst1 in mBM-MSCs exposed to H_2_O_2_ condition. **a** Cell viability was measured in mBM-MSCs treated with different H_2_O_2_ concentrations for 12 h by CCK-8 (*n* = 3). **b** Mst1 expression in mBM-MSCs treated with different concentrations of H_2_O_2_ for 12 h. **c**, **d** mBM-MSCs were transfected with Ad-LacZ or Ad-sh-Mst1, and the Mst1 expression was confirmed by qPCR and Western blot analysis (*n* = 3). **e** Effects of Mst1 inhibition on the cell viability of mBM-MSCs exposed to H_2_O_2_ condition. **f** Cell adhesion of mBM-MSCs/sh-Mst1 exposed to H_2_O_2_ condition (*n* = 3). Data are expressed as mean ± SD. **p < 0.01, *p < 0.05. H_2_O_2_, mBM-MSCs exposed to hydrogen peroxide only; H_2_O_2_ + Neg, mBM-MSCs transduced with adenoviruses harboring control vectors for Ad-sh-Mst1 (Ad-LacZ) followed by exposure to H_2_O_2_; H_2_O_2_ + sh-Mst1, mBM-MSCs transduced with adenoviruses expressing sh-RNA directed against Mst1 (Ad-sh-Mst1) followed by exposure to H_2_O_2_; H_2_O_2_ + sh-Mst1 + 3-MA, mBM-MSCs/sh-Mst1, and pretreated with 3-MA followed by exposure to H_2_O_2_

Cell viability and Mst1 expression was analyzed in the mBM-MSCs treated with different concentrations of H_2_O_2_ (50–500 μM) for 12 h. Cell viability (using CCK-8) decreased and Mst1 expression increased in a dose-dependent manner (Fig. 2a, b). Approximately 47.8% and 56.8% cell viability was decreased in H_2_O_2_ concentration of 250 μM and 200 μM, respectively (Fig. 2a). Thus, 250 μM of H_2_O_2_ was the concentration used in the experiments. Mst1 expression increased by approximately 178.5% in mBM-MSCs treated with 250 μM of H_2_O_2_ for 12 h (Fig. 2b).

Mst1 expression was then suppressed via Ad-sh-Mst1 transduction. The efficiency of adenovirus transfection was determined by qPCR and Western blot analysis. qPCR data indicated that the expression level of Mst1 decreased by approximately 57.8% in mBM-MSC/sh-Mst1 groups than in mBM-MSC groups and by approximately 41.3% in mBM-MSC/negative groups (Fig. 2c, *p* < 0.05). mBM-MSC/sh-Mst1 groups also exhibited a significantly decreased levels of Mst1 than mBM-MSCs groups (0.409 ± 0.032 vs. 0.693 ± 0.052; *p* = 0.0013) and mBM-MSC/negative groups (0.409 ± 0.032 vs. 0.708 ± 0.072; *p* = 0.0028) (Fig. 2d). The results of qPCR and Western blot analysis indicated that Mst1 was successfully suppressed in transfected mBM-MSCs.

The “homing” process consists of a series of interrelated steps, including cell attachment to distant organs [23]. Cell viability and cell adhesion decreased in H_2_O_2_ groups than in control groups (Fig. 2e, f, *p* < 0.01). Both increased in sh-Mst1 groups than in H_2_O_2_ groups (*p* < 0.01).

### Mst1 inhibition activated autophagy in H_2_O_2_-treated mBM-MSCs

**Fig. 3:**
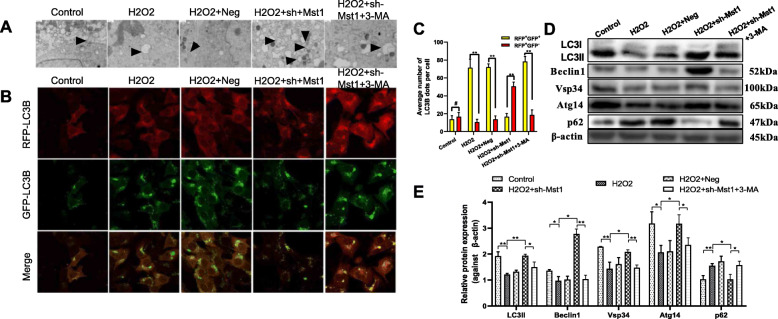
Mst1 inhibition activated autophagy in mBM-MSCs exposed to H_2_O_2_. The modulated mBM-MSCs were exposed to 200 μM H_2_O_2_ for 12 h. **a** Representative TEM photomicrographs showing the formation of autophagosomes containing organelle fragments (arrows). **b**, **c** mBM-MSCs transduced with stubRFP-sensGFP-LC3 lentivirus. The yellow puncta indicate autophagosomes, and the free red puncta indicate autolysosomes. At least 8–10 cells per condition were imaged. Quantification represents the ratio of autolysosome/autophagosome in each group. **p < 0.01, ^#^*p* > 0.05. **d**, **e** LC3-II/I, p62, Atg14, Beclin1, and Vsp34 expression levels were evaluated by Western blot analysis. β-actin was used as the loading control. Data are expressed as mean ± SD. **p < 0.01, *p < 0.05. H_2_O_2_, mBM-MSCs exposed to H_2_O_2_ only; H_2_O_2_ + Neg, mBM-MSCs/Neg followed by exposure to H_2_O_2_; H_2_O_2_ + sh-Mst1, mBM-MSC/sh-Mst1 followed by exposure to H_2_O_2_; H_2_O_2_ + sh-Mst1 + 3-MA, mBM-MSCs/sh-Mst1 pretreated with 3-MA followed by exposure to H_2_O_2_

Autophagy alterations were monitored by analyzing TEM to determine whether Mst1 inhibition induced autophagy (Fig. 3a). TEM images showed that the increased number of autophagic vacuoles (double membrane-bound autophagosomes) in sh-Mst1 groups compared with that in the H_2_O_2_ groups, thereby confirming the occurrence of autophagy and autophagosomes in mBM-MSC/sh-Mst1 exposed to H_2_O_2_. However, 3-MA also decreased autophagy in mBM-MSC/sh-Mst1.

The effects of Mst1 inhibition on autophagy flux was monitored via a tandem labeled GFP-mRFP-LC3B construct (Fig. 3b, c). The acid-sensitive green fluorescent protein (GFP) was quenched in the acidic environment of the lysosome, whereas mRFP was resistant. Therefore, fusing autophagosomes with lysosomes results in the loss of the yellow puncta and the appearance of a red-only puncta. As shown in Fig. 3, only parts of the LC3B-positive puncta were yellow in mBM-MSCs/sh-Mst1 exposed to H_2_O_2_ condition. Conversely, 3-MA inhibited autophagy, resulting in predominantly autophagosomes (yellow) in cells.

The effects of Mst1 inhibition on the expression of several critical autophagy-related proteins was also examined (Fig. 3d). Western blot analysis showed that Mst1 inhibition prevented the H_2_O_2_-induced significant downregulation of LC3 II/I, Beclin1, Vsp34, and Atg14 and p62 upregulation (*p* all < 0.05). By contrast, pretreatment with 3-MA reversed the above changes (*p* all < 0.05).

### Mst1 inhibition decreased oxidative stress in H_2_O_2_-treated mBM-MSCs

**Fig. 4:**
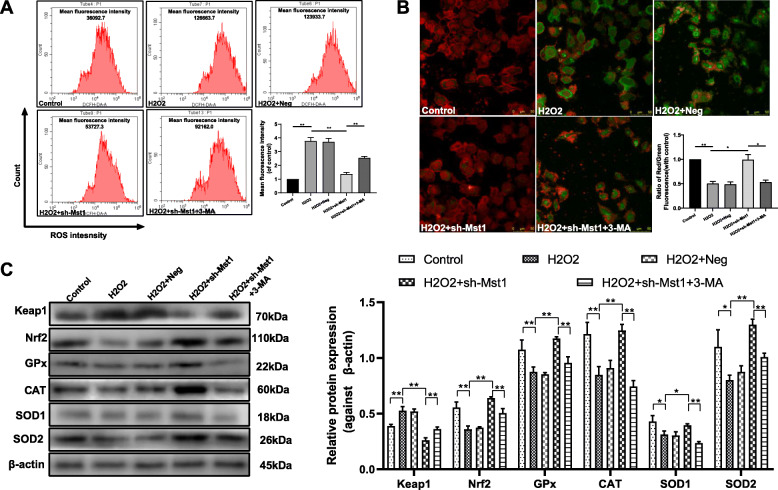
Mst1 inhibition attenuated H_2_O_2_-induced oxidative stress in mBM-MSCs. **a** Flow cytometry of ROS production in mBM-MSCs using the ROS probe DCFH-DA. (*n* = 3). Quantitative analysis of the intracellular ROS level. **p < 0.01. **b** Δψm was analyzed using JC-1 assay (*n* = 3). Decline in the membrane potential was reflected by the shift of fluorescence from red to green indicated by JC-1. Quantitative data of the red/green ratio (*n* = 3). **p < 0.01, *p < 0.05. **c** Keap1, Nrf2, GPx, CAT, SOD1, and SOD2 expression levels were evaluated by Western blot analysis. β-actin was used as the loading control. Data are expressed as mean ± SD. G, K. **p < 0.01, *p < 0.05. sh-Mst1 groups. H_2_O_2_, mBM-MSCs exposed to H_2_O_2_ only; H_2_O_2_ + Neg, mBM-MSCs/Neg followed by exposure to H_2_O_2_; H_2_O_2_ + sh-Mst1, mBM-MSC/sh-Mst1 followed by exposure to H_2_O_2_; H_2_O_2_ + sh-Mst1 + 3-MA, mBM-MSCs/sh-Mst1 pretreated with 3-MA followed by exposure to H_2_O_2_

ROS are important mediators of H_2_O_2_-induced cell death [24]. Intracellular ROS levels were assessed using the fluorescent probe DCFH-DA (Fig. 4a). The intracellular ROS levels in mBM-MSCs significantly increased following 250 μM H_2_O_2_ treatment for 12 h (*p* < 0.01). The increase in H_2_O_2_-induced ROS was blocked by Mst1 inhibition (*p* < 0.01). However, the decreased ROS production in mBM-MSCs/sh-Mst1 was reversed by pretreating cells with the autophagy inhibitor 3-MA (*p* < 0.01).

Δψm is a marker of mitochondrial statement. Δψm depletion in response to ROS insult initiates apoptotic cascades [25]. JC-1 is prone to aggregation in the mitochondrial matrix. When Δψm collapses, JC-1 disperses into monomers that convert fluorescence intensity from red to green. As expected, 250 μM H_2_O_2_ for 12 h treatment resulted in a noticeable reduction in Δψm of mBM-MSCs (Fig. 4b, *p* < 0.01), whereas Mst1 inhibition maintained the normally polarized Δψm as verified by fluorescence microscopy. In addition, the increased ratio of red/green fluorescence intensity was significantly decreased by 3-MA administration (*p* < 0.05).

Keap1, Nrf2, SOD, CAT, and GPx activities in H_2_O_2_-treated mBM-MSCs were measured to investigate whether Keap1/Nrf2 signaling activities were mediated by Mst1 inhibition (Fig. 4c). When mBM-MSCs were treated with 250 μM of H_2_O_2_, Nrf2, SOD, CAT, and GPx activities were significantly decreased compared with those of the control groups (*p* all < 0.05), whereas Mst1 inhibition significantly decreased the expression of Keap1, an inhibitor of Nrf2, compared with that of the H_2_O_2_ groups (*p* < 0.01). However, 3-MA suppressed the activated Keap1/Nrf2 signal in mBM-MSCs/sh-Mst1 exposed to H_2_O_2_ condition (*p* all < 0.01).

### Mst1 inhibition attenuated H_2_O_2_-induced cell apoptosis

**Fig. 5:**
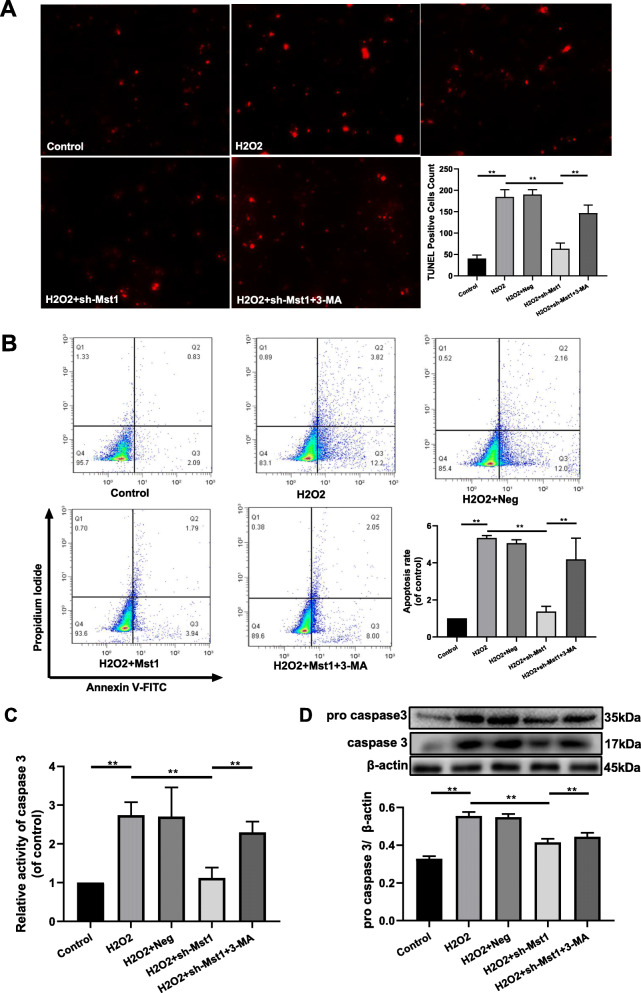
Cytoprotective effects of Mst1 inhibition toward H_2_O_2_-induced apoptosis in mBM-MSCs. Modulated mBM-MSCs were exposed to 200 μM of H_2_O_2_ for 12 h. **a** Representative images of TUNEL-positive mBM-MSC staining in the different groups. The content of TUNEL-positive cells was the number of green points in each image. Scale bar = 500 μm. **b** Cell apoptosis was analyzed by Annexin V-FITC/PI staining, detected by FACS (*n* = 3), and quantified on the basis of apoptosis rate (**d**) (*n* = 3). **c** Caspase 3 activity was measured by caspase 3 activity assay (*n* = 3). **d** Pro caspase 3 expression was analyzed by Western blot analysis. β-actin was used as the loading control. Data are expressed as mean ± SD. **p < 0.01, *p < 0.05. H_2_O_2_, mBM-MSC exposed to H_2_O_2_ only; H_2_O_2_ + Neg, mBM-MSC/Neg followed by exposure to H_2_O_2_; H_2_O_2_ + sh-Mst1, mBM-MSC/sh-Mst1 followed by exposure to H_2_O_2_; H_2_O_2_ + sh-Mst1 + 3-MA, mBM-MSCs/sh-Mst1 pretreated with 3-MA followed by exposure to H_2_O_2_

TUNEL-positive cells were detected by fluorescence microscopy to verify the effects of Mst1 inhibition on cell apoptosis (Fig. 5a). Data from the TUNEL assay showed that H_2_O_2_ significantly increased mBM-MSC apoptosis (*p* < 0.01), which was alleviated via Mst1 inhibition (*p* < 0.01). However, 3-MA treatment increased mBM-MSC/sh-Mst1 apoptosis after exposure to H_2_O_2_ (*p* < 0.01). Similar results were observed via Annexin V-FITC/PI staining analyzed by FACS (Fig. 5b, *p* all < 0.05). In line with the above findings, direct measurements of caspase 3 activity and expression of pro caspase 3 revealed that Mst1 inhibition blocked caspase 3 activity, which was reversed by pretreatment in mBM-MSCs exposed to H_2_O_2_ (Fig. 5c, d, *p* all < 0.01).

### Mst1 inhibition-stimulated Keap1/Nrf2 signaling pathway activation is involved in cytoprotection

**Fig. 6:**
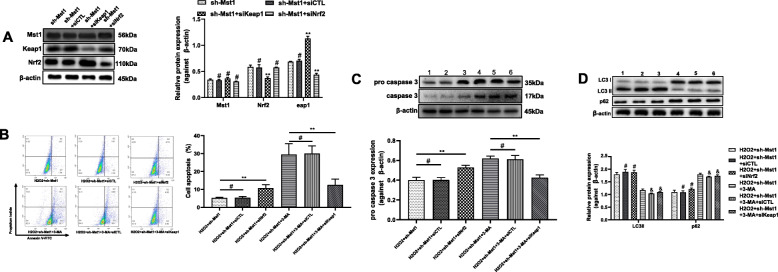
Mst1 inhibition activated the autophagy/Keap1/Nrf2 signaling pathway in mBM-MSC exposed to H_2_O_2_. mBM-MSC/sh-Mst1 was transfected with siCTL-siRNA, siKeap1, or siNrf2; Mst1, Keap1, and Nrf2 expression levels in mBM-MSCs/sh-Mst1 were determined by Western blot analysis (**a**, **p < 0.01 vs. sh-Mst1 groups, ^#^*p* > 0.05 vs. sh-Mst1 groups), pretreated with3-MA, and treated with 250 μM of H_2_O_2_ for 12 h. Cell apoptosis was detected by PI staining and pro caspase 3 expression analysis (**b** and **c**, **p < 0.01, ^#^*p* > 0.05), LC3B and p62 expression in mBM-MSCs/sh-Mst1 and mBM-MSCs/sh-Mst1 + 3-MA (**d**, ^#^*p* > 0.05 vs. H_2_O_2_ + sh-Mst1 groups, ^&^*p* > 0.05 vs. H2O2 + sh-Mst1 + 3-MA groups). β-actin was used as the loading control. Data were expressed as mean ± SD

As shown in Fig. 4, the Keap1/Nrf2 signaling pathway was activated by Mst1 inhibition. The role of this pathway in the protective effects of Mst1 inhibition on mBM-MSCs was investigated. For this purpose, Keap1 or Nrf2 was silenced by siKeap1 or siNrf2 in mBM-MSCs/sh-Mst1, respectively. Keap1 or Nrf2 silencing did not impair Mst1 inhibition (Fig. 6, *p* > 0.05). Compared with the expression of mBM-MSCs/sh-Mst1 groups, that of Keap1 decreased by approximately 0.63-fold in mBM-MSCs/sh-Mst1+siKeap1 groups (*p* < 0.01), and Nrf2 expression decreased by approximately 0.62-fold in mBM-MSCs/sh-Mst1+siNrf2 groups (*p* < 0.01). Nrf2 expression was increased by siKeap1 in mBM-MSCs/sh-Mst1 (*p* < 0.01).

mBM-MSC/sh-Mst1 apoptosis was evaluated by PI staining. Data suggested that silencing Nrf2 increased mBM-MSC/sh-Mst1 apoptosis after exposure to H_2_O_2_ (Fig. 6, *p* < 0.05). mBM-MSC/sh-Mst1+3-MA apoptosis was evaluated by the expression of pro caspase 3. Keap1 silencing decreased the cell apoptosis of mBM-MSC/sh-Mst1+3-MA exposed to H_2_O_2_. Autophagy was not affected by either Nrf2 silencing in mBM-MSC/sh-Mst1 following H_2_O_2_ insult or Keap1 silencing in mBM-MSC/sh-Mst1+3-MA following H_2_O_2_ insult. This finding was verified by the nonsignificant change in LC3 II/I and p62 expression (Fig. 6, *p* > 0.05). Cumulatively, these results suggest that the Keap1/Nrf2 signaling pathway exhibits protective effects on Mst1 inhibition toward H_2_O_2_.

## Discussion

In this study, mBM-MSCs were genetically modified by Mst1 shRNA adenovirus. Mst1 inhibition protected mBM-MSCs against H_2_O_2_-induced cell apoptosis via autophagy activation. The findings suggest that autophagy and Keap1/Nrf2 signaling pathway are involved in the protective action against oxidative stress injury, thereby indicating a new mechanism on how genes protect mBM-MSCs against oxidative stress.

MSCs are mainly trapped in the lungs via intravenous injection [26] and exert their therapeutic action through “homing” [2]. Improving the “homing” ability of MSCs can enhance the therapeutic action [27]. A previous study showed that improving cell adhesion enhanced the MSCs homing [23]. In our study, cell adhesion was promoted by mBM-MSCs/sh-Mst1, which indicated that Mst1 inhibition may enhance mBM-MSC homing ability under oxidative stress.

The Atg14–Beclin1–Vps34 complex plays an important part in autophagy activation. Atg14 bridges Beclin1 interaction with Vps34 complex, thereby mediating autophagosome formation [28]. Mst1 facilitated the accumulation of p62, a protein degraded by autophagy, and inhibited the Atg14–Beclin1–Vps34 complex activity and suppressed autophagy [29]. Our results showed that Mst1 inhibition decreased p62 expression but increased Atg14, Beclin1, and Vps34 expression, suggesting that autophagy was reactivated in mBM-MSCs/sh-Mst1 and may provide cytoprotective effects against H_2_O_2_.

The physiological levels of cellular ROS serve as the second messenger in the signaling pathways of stem cell proliferation and growth, whereas the pathological ROS levels contribute to MSC apoptosis and restrain their differentiation [4]. The increased ROS levels promoted the shortening of telomere length of MSCs, cell senescence, and decrease in cell differentiation [30, 31]. Excessive ROS damaged the mitochondria, thereby activating caspase 3, followed by MSC apoptosis [32, 33]. Our findings support the above results and emphasize that Mst1 inhibition confers protection by significantly suppressing the levels of ROS; increasing the activities of SOD, CAT, and GPx activities; suppressing the collapse of mitochondrial membrane potential; and decreasing the activation of caspase 3 via autophagy in mBM-MSCs exposed to H_2_O_2_. These results provide evidence on the apparent relationship among the antioxidant, autophagy, and cytoprotective effects of Mst1 inhibition.

Mst1 inhibition provided efficient cytoprotection for H_2_O_2_-induced mBM-MSC apoptosis by activating autophagy. Autophagy maintains the self-renewal and regenerative potential of stem cells and may resolve the poor survival of MSCs in response to oxidative stress [34, 35]. Mst1 promoted cardiomyocyte apoptosis via suppressing autophagy below the physiological levels under oxidative stress [36]. Given its regulating role for apoptosis and autophagy, Mst1 may be a new therapeutic target for preventing cardiomyocyte death [11, 29]. In this study, Mst1 inhibition attenuated autophagy suppression, whereas autophagy inhibition by 3-MA application blocked the protective effects of Mst1 inhibition. These results indicate that autophagy activation is involved in the protective effects of Mst1 inhibition on mBM-MSCs.

Loss of Mst gene diminished the self-protective effect of Keap1/Nrf2 axis against oxidative stress in macrophage [37]. In this study, Mst1 inhibition reactivated the Keap1/Nrf2 signaling pathway in mBM-MSCs exposed to H_2_O_2_. These findings are in line with the above concept, indicating that the Keap1/Nrf2 signaling pathway activation is involved in the protective effects of Mst1 inhibition in mBM-MSCs. This result is verified by the increased cell apoptosis in mBM-MSCs/sh-Mst1 transfected with si-Nrf2. However, the Mst1 inhibition-induced activation of autophagy was not suppressed by Nrf2 inhibition, and 3-MA-induced suppression of autophagy was not activated by Keap1 inhibition. The Keap1/Nrf2 signaling pathway was blocked by the autophagy inhibitor 3-MA. Keap1 was degraded by autophagy for the maintenance of redox homeostasis, and the integrity of the Keap1-Nrf2 signaling pathway was maintained by autophagy activation [38]. Thus, these results indicate that the Keap1/Nrf2 signaling pathway is involved in the Mst1 inhibition-induced activation of autophagy in mBM-MSCs.

## Conclusion

In summary, we demonstrated that Mst1 inhibition mediates the cytoprotective benefit of mBM-MSCs against H2O2 oxidative stress injury. The underlying mechanisms involve activating autophagy and the Keap1/Nrf2 signaling pathway. These findings present the efficient protective capacity of transplanted MSCs in PAH.

## Supplementary information

**Additional file 1.**

## Data Availability

All data generated or analyzed during this study are included in this published article and its supplementary information files.

## Abbreviations

BM-MSC: Bone marrow mesenchymal stem cell
PAH: Pulmonary arterial hypertension
ROS: Reactive oxygen species
Mst1: Mammalian Ste20-like kinase 1
CCK-8: Cell Counting Kit 8
TUNEL: Transferase-mediated dUTP nick-end labeling
Δψm: Mitochondrial membrane potential
Vsp34: PI3 Kinase Class III
SOD: Superoxide dismutase
CAT: Catalase
GPx: Glutathione peroxidase
Nrf2: Nuclear factor erythroid 2-related factor 2
