# Supplementary material for: Dissecting molecular mechanisms underlying H2O2-induced apoptosis of mouse bone marrow mesenchymal stem cell: role of Mst1 inhibition
Source: Stem Cell Res Ther. 2020 Dec 9;11:526. doi: 10.1186/s13287-020-02041-7 (PMC7724846; doi:10.1186/s13287-020-02041-7)
Supplement: Supplementary file 1 — Additional file 1. [file 13287_2020_2041_MOESM1_ESM.docx]

**SUPPLEMENTAL METHODS**

mBM-MSC culture

In brief, the femurs and tibia were immediately dissected from the mice, and the bone marrow was flushed out using a syringe containing the complete culture medium, alpha modification of Eagle’s medium (α-MEM; Gibco, USA) supplemented with 10% fetal bovine serum (Gibco), and 1% penicillin/streptomycin (Gibco). Cells were washed with phosphate-buffered saline (PBS), filtered (through 70 μm nylon mesh filter), and cultured with the complete culture medium in a humidified atmosphere of 5% CO_2_ at 37 °C.

**Cell adhesion**

Briefly, Matrigel was diluted with cold serum-free α-MEM at the ratio of 1:3, then added to 96-well plates (50 μl /well), and incubated with PBS containing 1% BSA in 37 ℃. Cells (2×10^4^ cells/ml) was plated on matrigel-coated wells before centrifuged (400 g ×, 2 min) and incubated at 37 ℃ in 5% CO_2_. After incubated for 20 min, the plate was vibrated twice (30 s/per each). Non-adherent cells were removed by washing with PBS, and the adherent cells were fixed with100 μl paraformaldehyde for 15 min, stained with crystal violet for 15 min, washed with distilled water, and added 2% SDS in each well. The optical density (OD) was measured at 590 nm using Multiskan MK3 microplate reader. The experiment was repeated for three times.

**SUPPLEMENTAL TABLES**

**Table S1**

| Antigen | Cat No. | Dilution | Manufacturer |
| --- | --- | --- | --- |
| CD90-FITC | [553013](https://www.bdbiosciences.com/cn/applications/research/stem-cell-research/cancer-research/mouse/fitc-rat-anti-mouse-cd902-30-h12/p/553013) | 1/100 | BD Biosciences PharMingen, CA, USA |
| CD73-PE | [550741](https://www.bdbiosciences.com/cn/applications/research/stem-cell-research/mesenchymal-stem-cell-markers-bone-marrow/mouse/positive-markers/pe-rat-anti-mouse-cd73-ty23/p/550741) | 1/100 | BD Biosciences PharMingen, CA, USA |
| CD29-PE | [562801](https://www.bdbiosciences.com/cn/applications/research/stem-cell-research/cancer-research/mouse/pe-hamster-anti-mouse-cd29-hm-1-1/p/562801) | 1/100 | BD Biosciences PharMingen, CA, USA |
| CD44-PE | [560569](https://www.bdbiosciences.com/cn/applications/research/t-cell-immunology/t-follicular-helper-tfh-cells/surface-markers/mouse/pe-cy7-rat-anti-mouse-cd44-im7/p/560569) | 1/100 | BD Biosciences PharMingen, CA, USA |
| CD34-FITC | 553733 | 1/100 | BD Biosciences PharMingen, CA, USA |
| CD45-FITC | [553079](https://www.bdbiosciences.com/cn/applications/research/stem-cell-research/cancer-research/mouse/fitc-rat-anti-mouse-cd45-30-f11/p/553079) | 1/100 | BD Biosciences PharMingen, CA, USA |
| SCA-1-FITC | [557405](https://www.bdbiosciences.com/cn/applications/research/stem-cell-research/hematopoietic-stem-cell-markers/mouse/positive-markers/fitc-rat-anti-mouse-ly-6ae-d7/p/557405) | 1/100 | BD Biosciences PharMingen, CA, USA |
| CD105-PE | [562762](https://www.bdbiosciences.com/cn/applications/research/stem-cell-research/hematopoietic-stem-cell-markers/mouse/positive-markers/pe-cf594-rat-anti-mouse-cd105-mj718/p/562762) | 1/100 | BD Biosciences PharMingen, CA, USA |
| Mst1 | ab51134 | 1/1000 | Abcam, USA |
| SQSTM1 (p62) | ab155686 | 1/1000 | Abcam, USA |
| LC3B | ab51520 | 1/1000 | Abcam, USA |
| Beclin1 | ab210498 | 1/1000 | Abcam, USA |
| Atg14 | 96752S | 1/1000 | Cell Signaling Technology |
| PI3 Kinase Class III (Vsp34) | 4263S | 1/1000 | Cell Signaling Technology |
| caspase-3 | ab13847 | 1/1000 | Abcam, USA |
| pro caspase-3 | ab32499 | 1/1000 | Abcam, USA |
| Keap1 | ab139729 | 1/1000 | Abcam, USA |
| Nrf2 | ab137550 | 1/1000 | Abcam, USA |
| GPx | ab108427 | 1/1000 | Abcam, USA |
| Catalase (CAT) | ab16731 | 1/1000 | Abcam, USA |
| superoxide dismutase 1 (SOD1) | ab13498 | 1/1000 | Abcam, USA |
| SOD2 | ab13534 | 1/1000 | Abcam, USA |

Table S2

| **Gene** | **Sequence** |
| --- | --- |
| sh-Mst1 | 5′-GCCCTCACGTAGTCAAGTATT-3′ |
| Nrf2-siRNA | 5′-UGAAAGCACAGCAGAAUUTT-3′ |
| Keap1-siRNA | 5′-ATATCTACATGCACTTCGGGG-3′ |
| scrambled siRNA (siCTL) | 5′-AUUGUAUGCGAUCGCAGAC-3′ |

Table S3

| **Gene** | **Sense** | **antisense** |
| --- | --- | --- |
| Mst1 | 5′- GGG TCC CAG TAG CCA AGA T-3′ | 5′- GAG GCA CCA CAT ACC ATT CA-3′ |
| GAPDH | 5′-TGA CGT GGA CAT CCG CAA AG -3′ | 5′- CTG GAA GGT GGA CAG CGA CG-3′ |
